# Constraint-Induced Movement Therapy Combined With Botulinum Toxin for Post-stroke Spasticity: A Systematic Review and Meta-Analysis

**DOI:** 10.7759/cureus.17645

**Published:** 2021-09-01

**Authors:** Mohammad Nasb, Sayed Zulfiqar Ali Shah, Hong Chen, Ahmed S Youssef, Zhenlan Li, Lamis Dayoub, Abdullah Noufal, Abdallah El Sayed Allam, Manal Hassanien, Ahmed Amine El Oumri, Ke-Vin Chang, Wei-Ting Wu, Martina Rekatsina, Felice Galluccio, Abdullah AlKhrabsheh, Ammar Salti, Giustino Varrassi

**Affiliations:** 1 Department of Rehabilitation Medicine and Physical Therapy, Tongji Hospital, Huazhong University of Science and Technology, Wuhan, CHN; 2 Department of Rehabilitation Medicine and Physical Therapy, First Hospital of Jilin University, Jilin University, Changchun, CHN; 3 Department of Physical Therapy, Albaath University, Homs, SYR; 4 Department of Orthopedic Surgery, Al-Assad University Hospital, Damascus University, Damascus, SYR; 5 Physical Medicine, Rheumatology and Rehabilitation, Tanta University Hospitals & Faculty of Medicine, Tanta University, Tanta, EGY; 6 Department of Rheumatology, Assiut University Hospital, Assiut, EGY; 7 Immunohematology Cellular Therapy, Medical School Oujda / Mohammed VI University Hospital of Oujda, Oujda, MAR; 8 Physical Medicine and Rehabilitation, National Taiwan University Hospital Bei-Hu Branch, Taipei, TWN; 9 Department of Physical Medicine and Rehabilitation, National Taiwan University Hospital, Bei-Hu Branch, Taipei, TWN; 10 Pain Management, Whipps Cross Hospital Barts Health National Health Service (NHS), London, GBR; 11 Medical-Geriatric Department, Rheumatology, University Hospital Azienda Ospedaliero Universitaria (AOU) Careggi, Florence, ITA; 12 King Abdullah University Hospital, Jordan University of Science and Technology, Amman, JOR; 13 Anesthesia and Pain Medicine, Cleveland Clinic, Abu Dhabi, ARE; 14 Research, Paolo Procacci Foundation, Roma, ITA

**Keywords:** botulinum toxin, cimt, mcimt, neuroplasticity, rehabilitation, spasticity, stroke

## Abstract

Stroke is considered one of the main causes of adult disability and the second most serious cause of death worldwide. The combination of botulinum toxin type A (BTX) with rehabilitation techniques such as modified constraint-induced movement therapy (mCIMT) has emerged as a highly efficient intervention for stroke patients to start synchronized motor function along with spasticity reduction. The current systematic review and meta-analysis were conducted in order to evaluate the available literature about the safety and efficacy of constraint-induced movement therapy (CIMT) combined with BTX in stroke patients with upper limb spasticity.

Searches were conducted on WoS (Web of Science), Ovid, EBSCO-ASC&BSC, and PubMed for identifying relevant literature published from 2000-2020. Randomized Controlled Trials (RCTs) and Quasi-experimental studies were considered for inclusion. Rayyan (systematic review tool) QCRI (Qatar Computing Research Institute) was used for independent screening of the studies by two reviewers. For risk of bias and study quality assessment, Cochrane risk of bias tool (RoB 2) and Physiotherapy Evidence Database (PEDro) scales were used. Cochrane review manager was used to carry out the meta-analyses of the included studies.

The search resulted in a total of 13065 references, of which 4967 were duplicates. After the title, abstract and full-text screening, two RCTs were deemed eligible for inclusion. Both the RCTs scored 8 on PEDro and were level evidence. The studies were heterogeneous. The findings of this meta-analysis in all the three joints post-stroke spasticity assessed on modified Ashworth scale (MAS) at four weeks post-injection aren’t statistically significant (elbow P-value 0.74, wrist P-value 0.57, fingers P-value 0.42), however, according to one of the included studies the therapeutic efficacy of the combination of BTX-mCIMT injection assessed at four weeks post-injection in wrist and finger flexors was promising.

The effectiveness of BTX-CIMT combination over conventional therapy (CT) for improving post-stroke spasticity still needs to be explored with long-term, multicenter rigorously designed RCTs having a good sample size. However, the BTX-CIMT combination is promising for enhancing motor function recovery and improving activities of daily living (ADLs).

## Introduction and background

Stroke is among the most serious diseases globally. It affects millions of people all over the world. It is associated with a high economic burden every year due to rehabilitation and pain [1,2]. Consequently, it is not only affecting the economies of various countries but also lowering the quality of life of their populations [1]. Over the past few decades, multiple treatment strategies were developed to alleviate this health problem, including rehabilitation and drug therapy.

Rehabilitation techniques include but are not limited to mirror therapy, virtual reality, robot-assisted rehabilitation and constraint-induced movement therapy (CIMT) [3-5]. CIMT represents a high-intensity and task-specific training after stroke, particularly for the upper extremities. Nevertheless, the broad adoption of CIMT post-stroke is hindered by the presence of significant spasticity [6,7].

Spasticity, a common complication of stroke, hinders the rehabilitation process substantially and requires practical and effective management [8]. Its management may include non-pharmacological treatment modalities such as shock waves therapy and pharmacotherapy with oral anti-spasticity drugs such as baclofen, dantrolene, benzodiazepines, or phenol and alcohol injections [9-13]. However, some medications are frequently accompanied by side effects such as muscle weakness and fatigue [14].

A highly promising therapeutic approach to post-stroke spasticity was the injection of botulinum toxin type A (BTX), which is associated with minor side effects. Although the effect of BTX injections in combination with CIMT showed a promising impact on arm motor function and reduced spasticity in a chronic stroke patient, this combination has not been fully elucidated thus far [15].

The combination of BTX injections and CIMT (BTX-CIMT) is possibly a promising approach for such an important issue. These injections can counterbalance the arms’ motor recovery along with managing the spasticity [16]. Besides, the use of BTX-CIMT in upper limb post-stroke spasticity management has resulted in significant improvements in the capacity and performance of the affected upper limb [15, 17].

This meta-analysis aimed to evaluate the available literature about the safety and efficacy of CIMT combined with BTX in stroke patients with upper limb spasticity. This will keep researchers, decision-makers, and healthcare practitioners up-to-date with the current evidence on the topic.

## Review

Methods

Search Strategy

The databases/platforms Web of Science (WoS), Ovid, EBSCO-Academic Source Complete(ASC) and Business Source Complete (BSC), and PubMed, were used for literature search and study identification. These databases were searched to identify relevant literature at the end of January 2021. The Ovid of Huazhong University of Science and Technology library is a platform for searching databases, like EMBASE, MEDLINE, LWW Medical Full-text Journal Database, and LWW medical eBooks (Updated: 2019-3-29). Medical Subject Heading (MeSH) terms and keywords identified on these databases for this study are used as following: PubMed (botulinum toxin type A, spasticity, stroke, stroke Rehabilitation: EBSCO-MEDLINE (botulinum toxins, type A, stroke, stroke Rehabilitation, muscle spasticity): Ovid (botulinum toxin A, stroke, spasticity, stroke): EMBASE (constraint-induced therapy, accident, botulinum, muscle spasticity). These MeSH terms and keywords were used during basic and advanced searches on the databases. A search was also performed without using the above PubMed MeSH terms because using those MeSH terms may restrict the search to a subset of PubMed citations only. Boolean phrases, truncation, and wild cards were used wherever necessary. The search filters applied were publication years (2001-2020), species (humans), and article type (randomized controlled trial, clinical trial, comparative study, journal article). Other sources for study identification were screening the reference lists of the included studies.

Inclusion and Exclusion Criteria

The study inclusion criteria were as follows: 1) Randomized Controlled Trials (RCTs) and quasi-experimental studies that studied the combined effects of constraint-induced movement therapy and botulinum toxin A; 2) Enrollment of adolescents and adults with post-stroke spasticity [modified Ashworth scale (MAS) 1-⩾3]; 3) Quantitative measurement of post-stroke spasticity before and after BTX-CIMT therapy.

Studies with lower limb spasticity, pediatric stroke, or traumatic brain injury were excluded. Moreover, single-arm studies, animal studies, ex vivo/in vitro studies, review studies, retrospective studies, case series, and case reports were also excluded.

Studies Selection

The study selection process comprised three screening phases: study title screening, abstract screening, and finally, full-text screening. There were two independent (MN, SZAH) reviewers on all three screening phases, with the corresponding author resolving conflicts or disagreements between both reviewers. The search results were uploaded to Rayyan QCRI (Qatar Computing Research Institute), Data Analytics (rayyan.qcri.org) for citation screening [18] by two independent reviewers. After screening the titles, the abstracts of the selected titles were screened, and finally, the full texts of the selected studies were retrieved for review.

Types of Intervention

The application of modified constraint-induced movement therapy (mCIMT) or CIMT or conventional rehabilitation after BTX injection.

Types of Participants

Patients with chronic hemiplegia having post-stroke spasticity in the upper extremities.

Types of Outcome Measures

The primary outcome measure was upper limb spasticity measured via MAS as our primary variable. Upper limb functional improvement was taken as the secondary variable [Motor Activity Log (MAL), Fugl-Meyer assessment (FMA), Barthel index (BI)].

Study Quality Assessment and Risk of Bias Assessment

Data extracted for external validity and reporting quality included study ID [author(s) name(s) with the year and reference], study design characteristics (data regarding experimental design and study groups, number of participants per group), participants details (diagnosis, spasticity, and functional level), intervention (muscle selection, and method of localization, dosage, frequency, volume, follow up), control (control type, intervention), primary and secondary outcome measures, results and information regarding dropouts were collected.

The Physiotherapy Evidence Database (PEDro) scale was used for study quality assessment [19]. Two independent reviewers (ASAY, ZL) performed the study quality evaluation; any conflicts/disagreements were resolved upon comparing the recorded data and discussion with a third author (MN). The PEDro scale has 11 quality assessment items (yes or no), of which 10 items are used for calculating the final PEDro score (0-10). Sackett’s levels of evidence were used for simplifying study quality levels, and an RCT was considered to be Level 1 (higher quality) if it scored ⩾6 on the PEDro scale and Level 2 if it scored <6 [20].

Study risk of bias was assessed by two independent reviewers (LD, HA) by Cochrane risk of bias tool (RoB 2) [21]. Every study was rated on RoB 2 for selection bias, detection bias, performance bias, outcome assessing, incomplete data, outcome reporting, and other threats to study validity identified by reviewers. The risk of bias was categorized as low risk of bias (the domain was considered adequate), high risk of bias (the domain was considered inadequate), or unclear risk of bias (not enough information for bias judgment). The RoB 2 excel tool was filled for individual studies, and results were entered into the desktop version of Cochrane Review Manager 5.3 [22].

Data Extraction

Two independent reviewers (AESA, DSA) performed data extraction; any conflicts/disagreements were resolved on discussion with the corresponding author and comparing the recorded data. The Microsoft data extraction form consisted of details of the included studies, design, population, target variables, interventional procedure, and results. Authors (HKM, AAEO, KVC, WTW) pre-piloted the data extraction form on some studies. Data were extracted from the texts, tables, and figures of the included studies.

Data Synthesis

We performed both qualitative and quantitative analyses. The data extracted were summarized in table form to present the characteristics and results of the included studies effectively. A meta-analysis was performed for the study primary variable using a random-effect model. We planned to do subgroup analysis and sensitivity analysis, but due to a lack of studies, we could not proceed with these analyses.

Meta-Analysis Methodology

Quantitative analysis was performed for the study's primary variable, spasticity (measured via MAS). The mean MAS changes in the elbow, wrist, and finger joints at four weeks post-intervention time points between the study groups were used to compare the effects of BTX-CIMT combination therapy with BTX A plus conventional rehabilitation approaches on post-stroke spasticity. For the outcome of interest, we calculated differences in means by the random effect model, with 95% confidence intervals (CI). An effect size of 0.2, 0.5, and 0.8 was considered a small, moderate, and large effect size, respectively [23]. The estimates of heterogeneity I^2^ index (I^2^) and Chi-square tests (Chi) were used for evaluating heterogeneity among the studies. We considered an I^2^ value of 25% as low, 50% moderate, and 75% as depicting high heterogeneity [24]. All the analyses were performed on Cochrane Review Manager 5.3 [22], and a p < 0.05 was considered statistically significant. The risk of bias in the included studies was assessed with the Cochrane Risk of Bias tool (RoB 2), while for the study quality appraisal, we used PEDro scale [19].

Results

Study Selection

**Figure 1:**
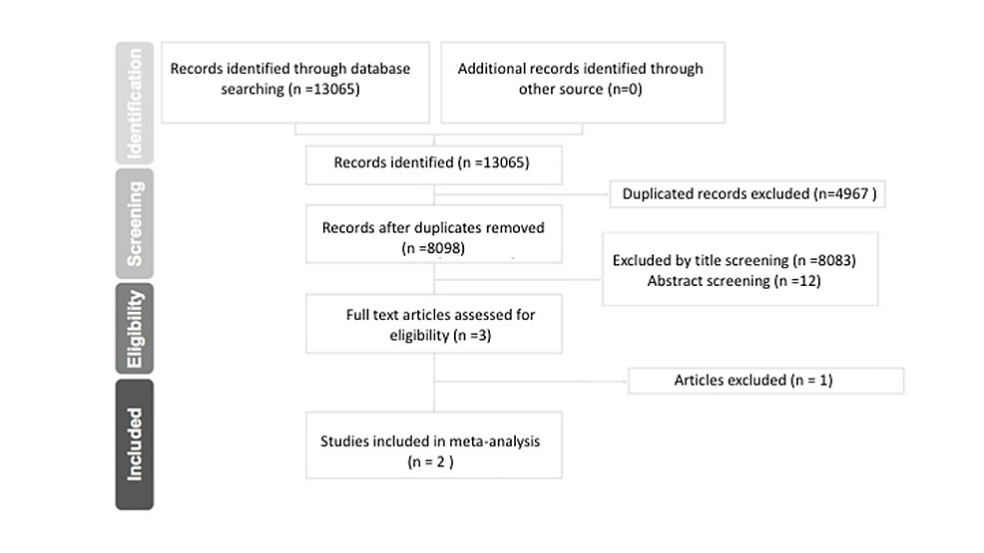
PRISMA flow diagram of search results PRISMA - Preferred Reporting Items for Systematic Reviews and Meta-Analyses

The search resulted in a total of 13,065 references, of which 4,967 were duplicates. After duplicates removal, 8098 articles were left, of which 8083 irrelevant studies were excluded during title screening, and 12 studies were excluded during the abstract screening because of the methodical design. One study was further excluded during full-text screening because of the study design. Only two studies were deemed eligible for inclusion. Both the studies were included in qualitative and quantitative synthesis (Figure 1). Both the included studies were RCTs [15, 17].

Study Characteristics

The final systematic review and meta-analysis included two RCTs having two study arms with a total of 93 participants. The studies included in the meta-analysis compared the combined effects of BTX-CIMT with the BTX plus conventional therapy (CT) for managing post-stroke spasticity and enhancing functional recovery.

Both of the studies enrolled participants with unilateral chronic hemiplegia. The participants' detail upon enrollment in the included studies are as follows: age 10 to 70 years, MAS score of ≥1 [17], age 18 to 80 years; MAS score of ≥3 in the elbow, wrist, or finger flexors [15].

The included studies used different brands and units of BTX, but both diluted BTX in 0.9% saline to acquire the target concentration. A high dose of 1,000 units per affected extremity of BTX prepared using Dysport® (Ipsen Ltd, Berkshire, UK) supplied in a 500-unit vial, diluted in 0.9% to get a concentration of 200 units/ml was used by one study [15]. However, the other study injected BTX from BOTOX® (AbbVie Inc., North Chicago, Illinois), which comes in 100-unit vials, added to 0.9% sterile normal saline (SNS) in the required concentration [17].

Both the included studies started the rehabilitation therapy one day after the BTX injection [15]. In one of them, BTX was injected by a trained physician under ultrasound guidance, following WHO guidelines [17]. In the other study, the injections were performed by a physician in the motor endplate zones using routine electromyography [15].

According to one study, a soft padded glove was used to restrain the unaffected limb [17]. The other study used a soft mitt as a non-affected arm restrainer [15].

The intervention regimen adopted by one of the included studies in the BTX-CIMT combination group included: massed practice, shaping (shaping consisted of task selection, graded tasks difficulty and complexity, verbal feedback, and movements assistance), a behavioral contract, and a daily treatment diary [15]. The other study also used a combination of BTX-CIMT including: placing, reaching lifting, and grasping activities with the challenge increased over time [17].

The therapies followed for the control group in both studies were as follows, conventional rehabilitation sessions of physiotherapy, sessions of occupational therapy, comprising of neurodevelopmental techniques, focused on relieving muscle tone and normalizing movements patterns to achieve the targets of improving stance, gait, dexterity, and exercise endurance in one study [15]. The other study used intensive conventional therapy: stretching and strengthening, activities of daily living (ADLs) training and functional tasks, neurodevelopmental techniques, like Bobath’s and Brunnstrom’s methods [17]. One study investigates the effects of BTX-CIMT, or rehabilitation for 4 weeks [17], while the other studied the effects for six months [15]. The use of MAS for spasticity was common in both the included RCTs. A detailed description of the included studies and treatment regimens is given in Table 1.

**Table 1:** Details of the included studies ICT - Intensive conventional therapy; mCIMT - Botulinum toxin-intensive conventional therapy (BTX-ICT): Modified constraint-induced movement therapy; CIMT - constraint-induced movement therapy; BTX A - Botulinum Toxin A; Botulinum Toxin A combined with intensive conventional therapy (BTX-CT); ADLs - Activities of Daily Living; WMFT - Wolf Motor Function Test; MAL - Motor Activity Log (MAL); BBT - Box and Blocks Test; FM-UE - Fugl–Meyer Assessment of Motor Function Upper Extremity subtest; HEP - Home Exercise Plan; FCR - Flexor Carpi Radialis; FCU - Flexor Carpi Ulnaris; FDS - Flexor Digitorum Superficialis; FU - Forced-use Therapy; ARAT - Action Research Arm Test; AOU - Amount of use; QOM - Quality of movement; PT - Physiotherapy; OT - Occupational therapy

Study	Study design	Study population	Sample size/Groups	Intervention dosage	Treatment start/duration	Outcome measures	Adverse effects	Result	conclusion
Nasb et al. 2019 [17]	RCT	Post-stroke Spasticity, Unilateral stroke, Diagnosis= <1-year Age 10-70	n=64 BTX-mCIMT(n=32) BTX-CT(n=32)	Single-dose Biceps brachii=200 units at two sites Others muscles one site 150 units/point mCIMT 1h/d, 6d/wk CT, 1 h/day, 6 times/wk	mCIMT or CT commenced one day after BTX and mCIMT 4 weeks CT 4 weeks.	MASS FMA BI	Not reported	Mass within group=P value <0.05) Mass between groups=Non-significant (P value > 0.05) FMA= significant (P value0.01) BI=significant (P value 0.02)	BTX-CIMT had higher therapeutic effects than BTX-CT on functional recovery and ADLs
Sun et al. 2010 [15]	RCT	Unilateral chronic stroke patients with upper extremity spasticity. Mean age, 58.7	n=32 BTX A + mCIMT (n=16) BTX A + conventional rehabilitation (n=16)	Biceps brachii at 2 sites (each site 200 units), Others muscles 150 units into each Massed practice,2 h/d, 3 d/wk,3m Restraining, 5 h/d/3 months conventional rehabilitation 1h PT, 1h OT, 3 d/wk, 3m	mCIMT and CT, 1 day after BTX A FU, 3 months	MAS MAL ARAT AOU scale QOM scale Satisfaction rate	No adverse events	Mass= significant (P value 0.019) ARAT= significant (P value .012) Satisfaction rate BTX A + mCIMT=86.7 Satisfaction rate Satisfaction rate BTX A + mCIMT=64.3	BTX and mCIMT combination is effective and safe for improving spasticity and motor function.

Outcomes

1. Effects of Combined BTX-CIMT or BTX with intensive conventional therapy (BTX-CT) on post-stroke spasticity

**Figure 2:**
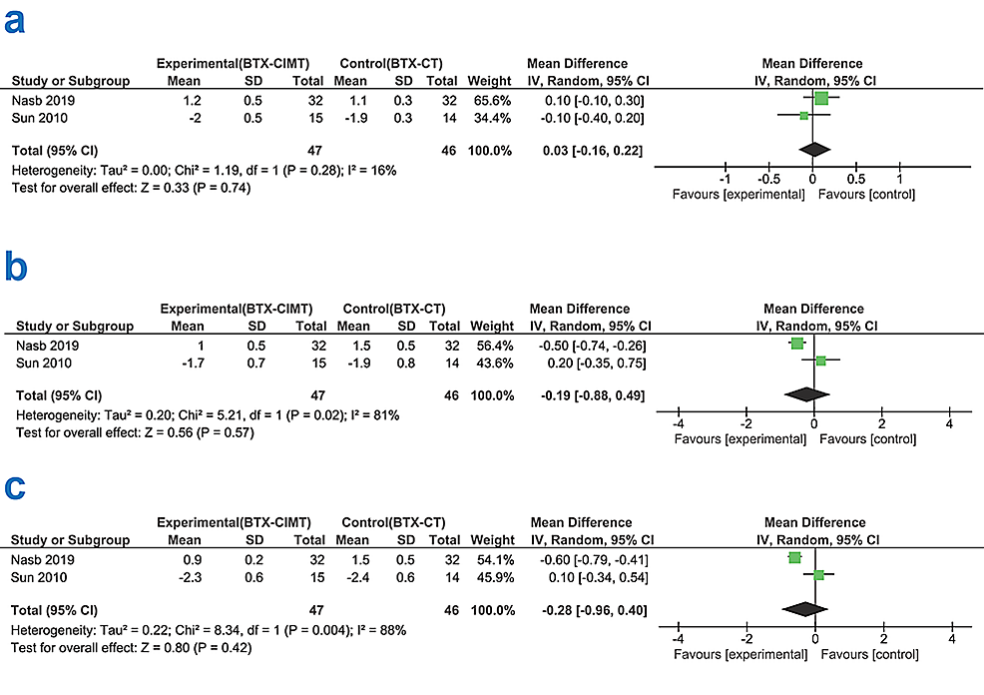
Results of the analysis of data extracted by the two examined publications (a) Shows Forest plot for comparison of the effects of combined BTX-CIMT or BTX-CT intervention on elbow joint spasticity by comparing mean differences using random-effect model, with 95% confidence interval. No statistical significance was identified (p-value 0.74) (b) Shows Forest plot for comparison of the effects of combined BTX-CIMT or BTX-CT intervention on wrist joint spasticity by comparing mean differences, using random-effect model, with 95% confidence interval. No statistical significance was identified (p-value 0.57) (c) Shows Forest plot for comparison of the effects of combined BTX-CIMT or BTX-CT intervention on finger joint spasticity by comparing mean differences, using random-effect model, with 95% confidence interval. No statistical significance identified (p-value 0.42) BTX-CIMT - Botulinum Toxin A - modified constraint-induced movement therapy; BTX-CT - Botulinum Toxin A - conventional therapy; Tau^2^ - Tau-squared; Chi^2^ - Chi-square test; df - Degree of freedom; I^2^ - I-squared index; IV = weighted mean difference; Z - z-statistics - significance test All data were collected from references [15,17].

The mean values of MAS for elbow, wrist, and finger joints from both the groups at four weeks post-intervention time points were used to study the combined effects of BTX-CIMT or BTX-CT on post-stroke spasticity. The studies showed substantial heterogeneity on the wrist (p-value 0.02, I^2^ 81%) and finger joints (p-value 0.004, I^2^ 88%) spasticity analysis but not in the elbow joint (p-value 0.28, I^2 ^16%). The findings of this meta-analysis for all the three joints assessed (elbow p-value 0.74, wrist p-value 0.57, finger p-value 0.42) are not statistically significant. However, the therapeutic efficacy of BTX-CIMT combination therapy for wrist and finger joints was judged promising in one of them [17]. More details are given in Figure 2 (a, b, c), including tau-squared (Tau^2^), which reflects the amount of the heterogeneity, the z-statistics - significance test for the weighted average effect size (Z), and the degree of freedom (df) which indicates the number of independent values that can vary in an analysis without breaking any constraints.

2. Adverse events

None of the studies reported any treatment-related adverse events during the course of treatment.

3. Risk of bias and study quality assessment

**Figure 3:**
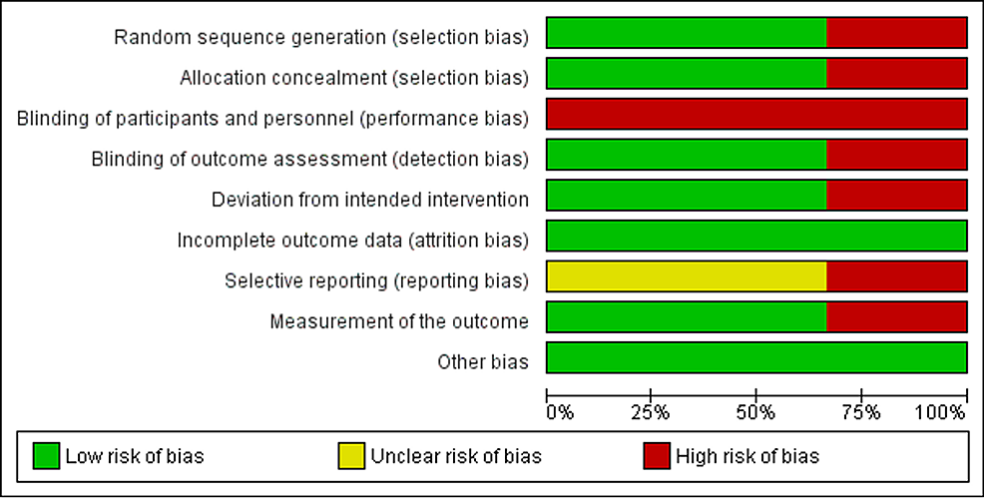
Shows RoB graph risk with bias items presented as percentages for all included studies References: [15,17,21] RoB - Risk of Bias

**Figure 4:**
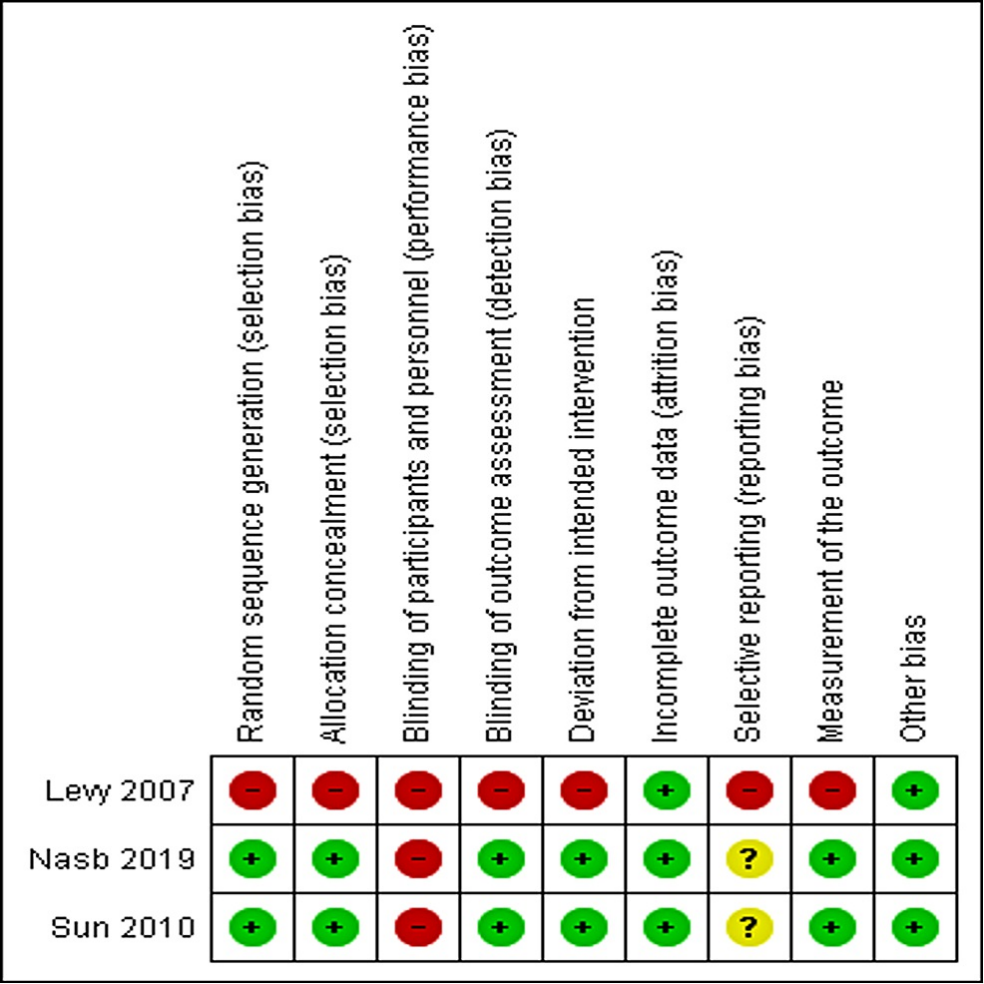
RoB summary: Review authors' judgements about each risk of bias item for the included studies. References: [15,17,21] RoB - Risk of Bias

None of the studies did blinding of patients and therapists, and were identified to have an unclear bias in reporting (Figures 3, 4).

Both the RCTs were level 1, higher-quality studies (PEDro score =8) (Table 2) [15,17]. 

**Table 2:** Quality of the included studies evaluated with PEDro Scale Reference: [21]

Study I. D	Item.1	Item.2	Item.3	Item.4	Item.5	Item.6	Item.7	Item.8	Item.9	Item.10	Item.11	Total score
Nasb.2019 [17]	Y	Y	Y	Y	N	N	Y	Y	Y	y	y	8
Sun-SF.2010 [15]	Y	Y	Y	Y	N	N	Y	Y	Y	Y	Y	8

Discussion 

Meta-Analysis Summary

This review included two RCTs to evaluate and compare the effectiveness of the combined application of BTX-CIMT or BTXA plus CT on the patients with post-stroke upper limb spasticity, providing an updated evidence base for policy-makers, physicians, and researchers. The findings of this meta-analysis in all the three joints post-stroke spasticity assessed on modified Ashworth scale (MAS) at 4 weeks post-injection are not statistically significant; however, according to one of the included studies, the therapeutic efficacy of BTX-CIMT combination therapy assessed at four weeks post-injection in wrist and finger flexors was promising as compared to BTX-CT combination [17].

Both the level 1 RCT studies included in this review provided within-group effectiveness of BTX-CIMT combination therapy, and all participants improved in their spasticity compared to their baseline results. The RCT study by Nasb et al. [17] reported no between-group effectiveness among BTX-CIMT and BTX-CT groups. In contrast, Sun et al. [15] reported significant between-group results in the elbow, wrist, and finger flexor muscles six months after injection. Both the studies showed significant improvements in motor functional activities and ADLs. The studies reported no adverse events related to treatment. However, the findings of the studies are limited because of the low number of studies eligible for meta-analysis and the heterogeneity in the included studies.

Both the RCTs started rehabilitation regimes one day after BTX therapy; however, there were still some interventional and sample size differences that could affect the study results. Nasb et al. [17] inclusion criteria for post-stroke spasticity were MAS score of ≥1, while Sun et al. [15] criteria were MAS score ≥ 3. Nasb et al. [17] used a low dose of BTX, whereas Sun et al. [15] used a high BTX dose. Nasb et al. [17] recruited 64 participants/ 32/ group, whereas Sun et al. [15] recruited 32/16/group, but only 29 completed the study. The small sample size in this last study makes the study findings less reliable. Nasb et al. provided therapy for only four weeks, while in post neurological spasticity, a longer treatment course could have given significant results [17].

There were slight differences in the treatments to control and treated groups in the 2 studies. One of them treated the randomized experimental groups with a massed practice consisting of placing, reaching lifting, and grasping activities of the affected arm while unaffected are restrained [17]. The other one used shaping (task selection, graded tasks difficulty and complexity, verbal feedback, and movements assistance), a behavioral contract, while nonaffected upper extremity was restrained with mitt/glove [15]. For the control group, rehabilitation therapy included stretching and strengthening, ADLs training and functional tasks, neurodevelopmental techniques, like Bobath’s and Brunnstrom’s methods in one study [17]. The other study used physiotherapy techniques and occupational therapy, and neurodevelopmental techniques [15]. The control groups treatments mostly focused on relieving muscle tone and normalizing movements patterns to achieve the targets of improving normalizing muscle tone, movement patterns, dexterity, and training endurance.

The significant improvement in motor functions and recovery reported in the qualitative analysis is consistent with other studies. One of the included studies reported significant improvements in the elbow, wrist, and finger flexors spasticity after six months, after combined BTX-CIMT as compared to combined BTX A injection and CT [15].

Levy et al. [25] reported modest gains with CIMT but the study had only four participants in CIMT plus home-exercise group and also had dropouts in those four participants. The CIMT participants reported improvements in the box and blocks test (BBT), the motor activity log (MAL), and the Wolf motor function test (WMFT) compared with their own baseline score. Unfortunately, the gains achieved during CIMT reversed 24 weeks 24 after, with a return of spasticity.

A case study with a one-year long-term follow-up reported that CIMT after BTX A injection resulted in improvement of outcome measures MAS, FA, ARAT significantly before and after one year of treatment [26].

Although many case studies and non-randomized studies had reported the superiority of BTX A and mCIMT or CIMT combination therapy, due to heterogeneity in the included studies as shown in this meta-analysis and the low number of studies included, we could not provide some guidance regarding the efficacy of BTX-CIMT combination as compared to conventional rehabilitation for managing post-stroke spasticity. However, the BTX-CIMT combination has proved to be highly effective for improving and enhancing functional performances and ADLs. The scores of the different scales such as FMA, MAL, BI, BBT, WFMT, and ARAT were found to be significantly improved with the BTX-CIMT combination as compared to conventional rehabilitation.

Study Limitations

This study has some limitations. The first limitation is the follow-up duration of the included studies; one study has a follow-up period of four weeks [17], while the other study has six months follow-up period [15]. The second limitation is the upper limb spasticity level at the time of enrollment (MAS score of ≥1 in Nasb et al. study [17], MAS score of ≥3 in Sun et al. study [15]). The third limitation is MAS as the only common assessment tool between the two included studies. The final limitation is the low number of studies for possible inclusion and the heterogeneity between the included studies, which has decreased the power of the conclusion derived from this meta-analysis.

## Conclusions

The effectiveness of the BTX-CIMT combination over conventional therapy for improving post-stroke spasticity still needs to be explored with long-term, multicenter rigorously designed RCTs having a good sample size. However, the BTX-CIMT combination is promising for improving motor function recovery and ADLs. Moreover, merging BTX-CIMT with other traditional modalities, such as functional electrical stimulation, can lead to a substantial effect on the improvement of chronic upper limb spasticity. Most BTX-CIMT trials have not addressed treatment protocols or therapy dosage.
